# Complete mitochondrial genome of the edible Basidiomycete mushroom *Thelephora aurantiotincta* (Aphyllophorales: Thelephoraceae) from China

**DOI:** 10.1080/23802359.2020.1869620

**Published:** 2021-02-20

**Authors:** Xu-Hui Chen, Wei-Wei Meng, Rong-Cui Liu, Yu-Xin Bai, Hao-Qi Xu, Rui Ding, Shi-Cheng Shao

**Affiliations:** a College of Bioscience and Biotechnology, Shenyang Agricultural University, Shenyang, China; b College of Land and Environment, Shenyang Agricultural University, Shenyang, China; c Gardening and Horticulture Department, Xishuangbanna Tropical Botanical Garden, Chinese Academy of Sciences, Mengla, China

**Keywords:** Mitochondrial genome, phylogenetic analysis, *Thelephora aurantiotincta*, Thelephoraceae

## Abstract

The complete mitochondrial genome of *Thelephora aurantiotincta*, an edible Basidiomycete mushroom species with ecological and economic value is reported in this study. The whole genome is a circular molecule 50,672 bp in length and encodes 42 genes as follows: 15 protein-coding genes, two rRNA genes and 25 tRNA genes. The A, T, C, G contents in the genome are 35.60%, 35.31%, 13.89%, and 15.20%, respectively. Phylogenetic analysis revealed a close relationship between *T. aurantiotincta* and *T. ganbajun*. This is the first complete mitochondrial genome for *T. aurantiotincta* that will be useful for providing basic genetic information for this important species.

*Thelephora* species (Aphyllophorales, Thelephoraceae) usually form ectomycorrhizal associations with trees (Yagame and Maekawa 2019) and contribute significantly to plant health and ecosystem stability (Ramírez-López et al. 2015). *Thelephora aurantiotincta* Corner 1968 is a very common *Thelephora* species with a wide distribution in the Yunnan province of China. It is mainly distributed in braodleaf-conifer forest and coniferous forest, and harvested as a healthy and edible mushroom for its unique flavor (Norikura et al. 2011; Li 2016). In this study, we assembled and characterized the complete mitochondrial genome of *T. aurantiotincta* for the first time in order to provide genetic information for this edible mushroom. The annotated mitochondrial genome sequence was submitted to GenBank under the accession number of MT234668.

The sample of *T. aurantiotincta* was purchased in the Yunnan Mushuihua wild edible mushroom market in Kunming, China (N25°0′, E102°43′). Total genomic DNA was extracted and stored in Yunnan Agricultural University, Yunnan, China (contact person SC Shao, and email shaoshicheng@xtbg.org.cn) with the archival number of MG58. The DNA was sequenced on the Illumina Hiseq 4000 sequencing platform by Shanghai Personal Biotechnology Co. Ltd, China. Clean pair-end reads were filtered and assembled using SPAdes v.3.11.0 software (Bankevich et al. 2012) to yield the complete mitochondrial genome. Genes were annotated using the online GeSeq-Annotation of Organellar Genomes program (Tillich et al. 2017) (https://chlorobox.mpimp-golm.mpg.de/geseq.html) based on the *T. ganbajun* mitogenome (KY245891). The tRNA structural predictions were made using RNAstructure Web Servers for RNA Secondary Structure Prediction (http://rna.urmc.rochester.edu/RNAstructureWeb/) and tRNAscan-SE v2.0.5 (Lowe and Chan 2016).

The complete mitochondrial genome of *T. aurantiotincta* is 50,672 bp in length with the A, T, C, G contents of 35.60%, 35.31%, 13.89%, and 15.20%, respectively. A total of 42 genes were annotated, including 15 protein-coding genes, two rRNA genes and 25 tRNA genes. Three of the tRNA genes (trnI, trnL, trnF) are duplicated, and two (trnM and trnS) occur in triplicate in the mitochondrial genome. One protein-coding gene, cox1, has an intron. All the tRNA genes can be folded into classical cloverleaf secondary structures. The genetic compositions and coding sequences are similar to other mushroom species in *Thelephora* (Wang et al. 2017).

The phylogenetic relationships of *T. aurantiotincta* and sixteen other Agaricomycetes genomes were evaluated based on protein-coding genes. The phylogenetic tree was constructed using 1000 non-parametric bootstrap replicates with the maximum-likelihood analysis in MEGA 7.0 (Kumar et al. 2016) designating *Phakopsora pachyrhizi* as the outgroup taxon. The analysis found that the mitochondrial genome of *T. aurantiotincta* was fully resolved in a clade with *T. ganbajun* (Figure 1). This is the first time to report the genetic relationship between *Thelephora* species at the molecular level, and the newly reported mitochondrial genome of *T. aurantiotincta* will provide valuable genetic information for species evolution and phylogenetic analysis of *Thelephora* and Thelephoraceae.

**Figure 1.**
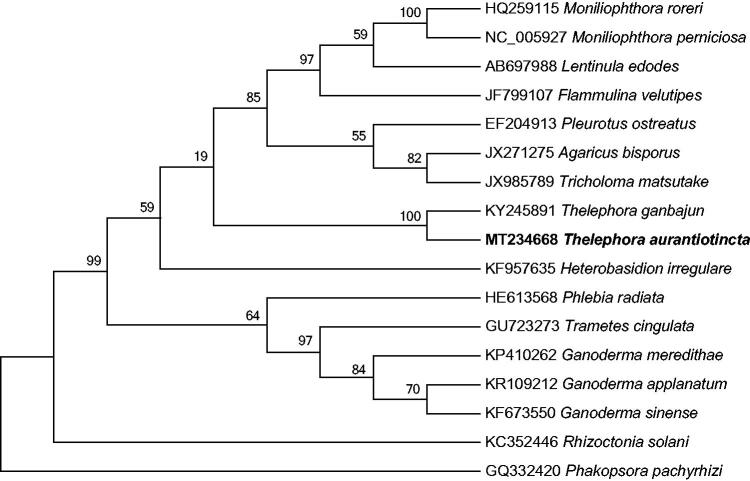
Phylogenetic relationship of *Thelephora aurantiotincta* and sixteen related species based on mitochondrial genome sequences. The phylogenetic tree was constructed using maximum-likelihood method based on the protein-coding genes, with *Phakopsora pachyrhizi* as the outgroup. The branch support was determined by computing 1000 non-parametric bootstrap replicates.

## Data Availability

Mitogenome data supporting this study are openly available in GenBank at nucleotide database, https://www.ncbi.nlm.nih.gov/nuccore/MT234668, Associated BioProject, https://www.ncbi.nlm.nih.gov/bioproject/PRJNA392534, BioSample accession number at https://www.ncbi.nlm.nih.gov/biosample/SAMN07303038 and Sequence Read Archive at https://www.ncbi.nlm.nih.gov/sra/SRR5803911.
